# Integrative Genome-Wide Association Studies of eQTL and GWAS Data for Gout Disease Susceptibility

**DOI:** 10.1038/s41598-019-41434-4

**Published:** 2019-03-21

**Authors:** Meng-tse Gabriel Lee, Tzu-Chun Hsu, Shyr-Chyr Chen, Ya-Chin Lee, Po-Hsiu Kuo, Jenn-Hwai Yang, Hsiu-Hao Chang, Chien-Chang Lee

**Affiliations:** 1 Department of Emergency Medicine, National Taiwan University Hospital, Taipei, Taiwan; 2 Department of Public Health and Institute of Epidemiology and Preventive Medicine, College of Public Health, National Taiwan University, Taipei, Taiwan; 3 Research Center for Genes, Environment and Human Health, National Taiwan University, Taipei, Taiwan; 4 National Center for Genome Medicine, Institute of Biomedical Sciences, Academia Sinica, Taipei, Taiwan; 5 Department of Pediatrics, National Taiwan University Hospital, National Taiwan University, College of Medicine, Taipei, Taiwan

## Abstract

There is a paucity of genome-wide association study on Han Chinese gout patients. We performed a genome-wide association meta-analysis on two Taiwanese cohorts consisting of 758 gout cases and 14166 controls of Han Chinese ancestry. All the participants were recruited from the Taiwan Biobank. For pathway analysis, we applied ICSNPathway (Identify candidate Causal SNPs and Pathways) analysis, and to investigate whether expression-associated genetic variants contribute to gout susceptibility, we systematically integrated lymphoblastoid expression quantitative trait loci (eQTL) and genome-wide association data of gout using *Sherlock*, a Bayesian statistical frame-work. In the meta-analysis, we found 4 SNPs that reached genome-wide statistical significance (*P* < 5.0 × 10^−8^). These SNPs are in or close to *ABCG2*, *PKD2* and *NUDT9* gene on chromosome 4. ICSNPathway analysis identified rs2231142 as the candidate causal SNP, and *ABCG2* as the candidate gene. Sherlcok analysis identified three genes, which were significantly associated with the risk of gout (*PKD2*, *NUTD9*, *and NAP1L5*). To conclude, we reported novel susceptible loci for gout that has not been previously addressed in the literature.

## Introduction

Gout is a common inflammatory arthritis that results from the deposition of monosodium urate crystals in joints. Epidemiological studies from a range of countries suggest a high prevalence of gout. Gout affects more than 3 million adults in the United States, and over 700,000 adults in the United Kingdom (UK)^1–3^. In the UK, gout is a common inflammatory joint disease affecting 2.5% of the population in 2012, with prevalence as high as 14% in men aged over 75 years^4^.

A prerequisite for gout development is hyperuricaemia, which is caused by an imbalance in the rates of production and excretion of uric acid. Most gout patients have hyperuricaemia and a clear concentration-dependent relationship exists between serum urate concentrations and incident gout^5^. The familial nature of hyperuricaemia and gout has been recognized since 17th century^6^. Genome-wide data in Europeans estimate that the heritability of serum urate levels was 27–41% and heritability of gout was approximately 30%^7,8^. The overall pattern of inheritance is best explained by a complex model incorporating interactions between more than one major gene, several modifying genes and environmental factors.

Genome-wide association studies (GWASs) have explored many genes associated with gout, for instance, *ABCG2*, *PKD2*, *SLC2A9*, *KCNQ1*, *SLC22A12* and *SLC17A1* for gout disease among individuals of European descent^9–12^. However, the genetic studies conducted to date have largely been restricted to patients of European ancestry, and there were limited GWAS studies in the Han Chinese population^13,14^. Therefore, conducting a GWAS study in the Han Chinese population may contribute to the understanding of the genetic causes of gout.

The standard data analysis of GWAS is based on a single SNP and may ignore the combined effect of modest SNPs/genes. To solve this problem, pathway-based analyses have been developed to extract more biological information from existing GWAS datasets. The ICSNPathway (identify candidate causal SNPs and pathways) analysis has been developed to identify candidate SNPs and their corresponding candidate pathways using GWAS data and by integrating linkage dis-equilibrium (LD) analysis, functional SNP annotation, and pathway-based analysis^15^. Thus, the integrative analysis using ICSNPathway might provide new insights for the understanding on the genetic basis of gout.

In addition, recent studies have used integrative strategies to combine results from association studies and eQTL (expression quantitative trait loci) analyses to interrogate the potential regulatory effect of the susceptibility SNPs in GWAS. He *et al*. developed a tool called Sherlock to systematically explore the role of a gene in complex diseases by integrating not only eQTL cis- but also trans-effects of that gene in GWAS^16^. This tool has been found to uncover many new susceptible genes that cannot be identified using GWAS alone in different diseases such as Crohn’s Disease, schizophrenia, and psoriasis^16–18^. As far as we were aware of, an integrative analysis of lymphoblastoid eQTL and GWAS data for gout disease susceptibility has not been conducted in Han Chinese. Hence, one aim of this research is to explore susceptibility genes in lymphocytes with regulatory function in gout by using Sherlock.

Therefore, the aim of this study is three-fold. The first aim was to identify genetic loci related to gout using GWAS in the Han Chinese population in Taiwan. The second aim is to conduct pathway analysis using the ICSNPathway method, to identify SNP and pathways related to gout. Third, we aimed to explore susceptibility genes in lymphocytes with regulatory function in gout by using Sherlock, a tool that integrates not only eQTL cis but also trans-effects of that gene in GWAS.

## Methods

### Study population

This study incorporated 15,300 Taiwanese Han subjects randomly selected from the Taiwan Biobank. Taiwan Biobank is a population-based biomedical research database that has collected detailed health and lifestyle information on participants^19,20^. Inclusion criteria were individuals who were aged between 30–70 years old and self-reported as being of Taiwanese Han Chinese ancestry. Patients diagnosed with cancer were excluded. In addition, aboriginal people and descents of foreigners were excluded to avoid population substructure. According to a recent study investigating the population admixture of Han Chinese residing in Taiwan, a high homogeneity was demonstrated among the Taiwanese subpopulations^20^.

### Study Variables

Participants of Taiwan Biobank were asked to fill a detailed questionnaire form. The detailed questionnaire form contained information on demographics, and previous medical history. Gout cases were identified from the self-reported questionnaire form, which has been evidenced to be the best test performance characteristics of existing definitions with sensitivity 80% and specificity 72%^21^. Controls were those without self-reported gout.

### Genotyping and quality controls

Whole genome genotyping was performed using the customized Axiom-Taiwan Biobank Array Plate (TWB chip; Affymetrix Inc, CA, USA) for both the GWAS and replication samples. Containing 653, 291 SNPs, TWB chip was designed to screen SNPs in genome-wide scale especially for Han-Chinese descent in Taiwan. The genotype information and linkage disequilibrium (LD) of healthy subjects have been released by the ethic and governance council of Taiwan Biobank (TaiwanView: http://taiwanview.twbiobank.org.tw).

Quality control procedures were done using plink with each individual, including gender concordance, sample quality, kinship, and population stratification (Supplementary Table 1). We did not observe any participant with sex mis-match for the discovery sample. No participants were removed at a call rate >0.97. However, when we searched for close relatives using identity-by-descent (IBD), 206 individuals with strong kinship (IBD > 0.8) were eliminated. To evaluate potential stratification in our study population, we also performed a principal component analysis (PCA). We identified no outliers from the scatter plot (Supplementary Figure 1). As a result, 7094 subjects, including 373 gout patients and 6721 healthy controls were retained. For the follow-up sample, following the same quality control procedures for individuals, 170 subjects were removed, resulting in a total of 7830 subjects, including 385 cases and 7445 controls. Quality control was also performed for SNPs. We removed markers if they failed Hardy-Weinberg tests with *P* < 0.0001, genotype missing rate >5%, and minor allele frequency (MAF) < 0.05. As a result, a total of 631,941 SNPs in the discovery samples, and 621,874 SNPs in the follow-up samples were retained (Supplementary Table 2).

### Statistical Analyses

#### GWAS analysis

Using 607,675 SNPs after quality control, the association of SNPs with the phenotype was tested by multivariate logistic regression analysis with adjustment for age at recruitment, gender, and the first 10 principal components. Odds ratios were calculated by considering the non-risk allele as a reference. We determined the minimum *P* value under three genetic models (additive, recessive and dominant). Ten principal components were included as covariates in the logistic regression model to control for population stratification, although genomic inflation was acceptable (<1.006) even before this correction was applied. The genomic inflation factor was derived by applying *P* values from logistic regression in an additive model for all the tested SNPs. A quantile–quantile plot of GWAS was used to examine the P-value distribution (Supplementary Fig. 2).

We decided to use the significance threshold of *P* = 5.0 × 10^−8^ in the fixed effect meta-analysis combining both discovery and follow-up sample. Power analysis can be found in Supplementary Table 3. Heterogeneity among the studies was determined by Cochrane’s Q statistic. LocusZoom plots were created using the LocusZoom tool (found at http://locuszoom.sph.umich.edu/locuszoom/) and the “hg19/1000 Genomes Nov 2014 ASN” panel was selected^22^. For general statistical analysis, we used R statistical environment version 3.51 or PLINK version 1.9. This research project was approved by the ethics committee of National Taiwan University Hospital Institutional Review Board. The study was conducted in accordance with the principles of the Declaration of Helsinki and the Good Clinical Practice Guidelines, and all the participants were informed consent.

#### ICSNPathway using GWAS data

We applied ICSNPathway analysis to the full list of gout GWAS SNPs p value^15^. ICSNPathway analysis involves two stages: (1) SNP clumping, which pruned SNPs by LD while prioritizing by p-value; (2) annotation of the biological mechanisms to pre-selected candidate SNPs using a pathway-based algorithm named i-GSEA (improved-gene set enrichment analysis). To avoid stochastic bias and the testing to general biological processes, we discarded pathways that contained <5 or >20 genes.

#### Sherlock

Using the web-based tool Sherlock, we implemented the integrated analysis of GWAS data and public lymphoblastoid eQTL data^16^. Lymphoblastoid B cells are selected as these cells are involved in the acute stage of gout. The underlying assumption is that the expression level of a specific gene(s) may influence the risk of a disease (eg, gout). Therefore, genetic variation (both in *cis*, and in *trans*) that perturbs gene expression may affect the risk of this disease. *Sherlock fi*rst searches for all eSNPs of each gene using the whole genome eQTL data from lymphoblastoid B cells. For each eSNP, *Sherlock* will then evaluate its association with gout using genome-wide association (GWA) data of gout. There can be three scenarios: (1) If the eSNP of a specific gene is also associated with gout in GWAS, a positive score would be given; (2) If the eSNP of this gene is not associated with gout, a negative score would be assigned; and (3) association only in GWAS (ie, non-eSNPs) does not alter the score. The total score of a gene increases along with the increase in the number of SNPs with combined evidence. For each gene, Sherlock performs a Bayesian inference to test whether the expression change of this gene has any impact on the risk of gout by using the collective information of the putative eSNPs of the gene. Based on the combined evidence from GWAS and lymphoblastoid eQTL, Sherlock infers gout-associated genes by calculating the logarithm of the Bayes factor of each gene. Compared with traditional analysis, which usually ignores SNPs with a moderate association (e.g., SNPs with P-values ranging from 1 × 10^−6^), Sherlock utilizes both strong and moderate SNPs in the eQTL and GWAS data through using a powerful statistical model. Sherlock makes the statistical inference by aggregating the information from both strong SNPs and moderate SNPs (strong SNPs have a larger contribution to the final score).

## Results

**Figure 1:**
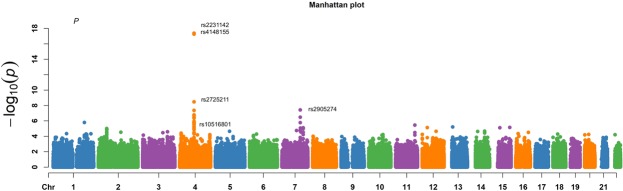
Manhattan plots for genome-wide SNPs associated with gout. Results of genome-wide association analysis (−log_10_ P) shown in chromosomal order for 631, 941 SNPs tested for association in initial sample of 373 cases and 6721 controls. The x axis represents each of the SNPs used in the primary scan. The y axis represents the −log10 P-value obtained by logistic regression analysis (additive model) with adjustment for age, gender, and 10 principal components.

**Figure 2:**
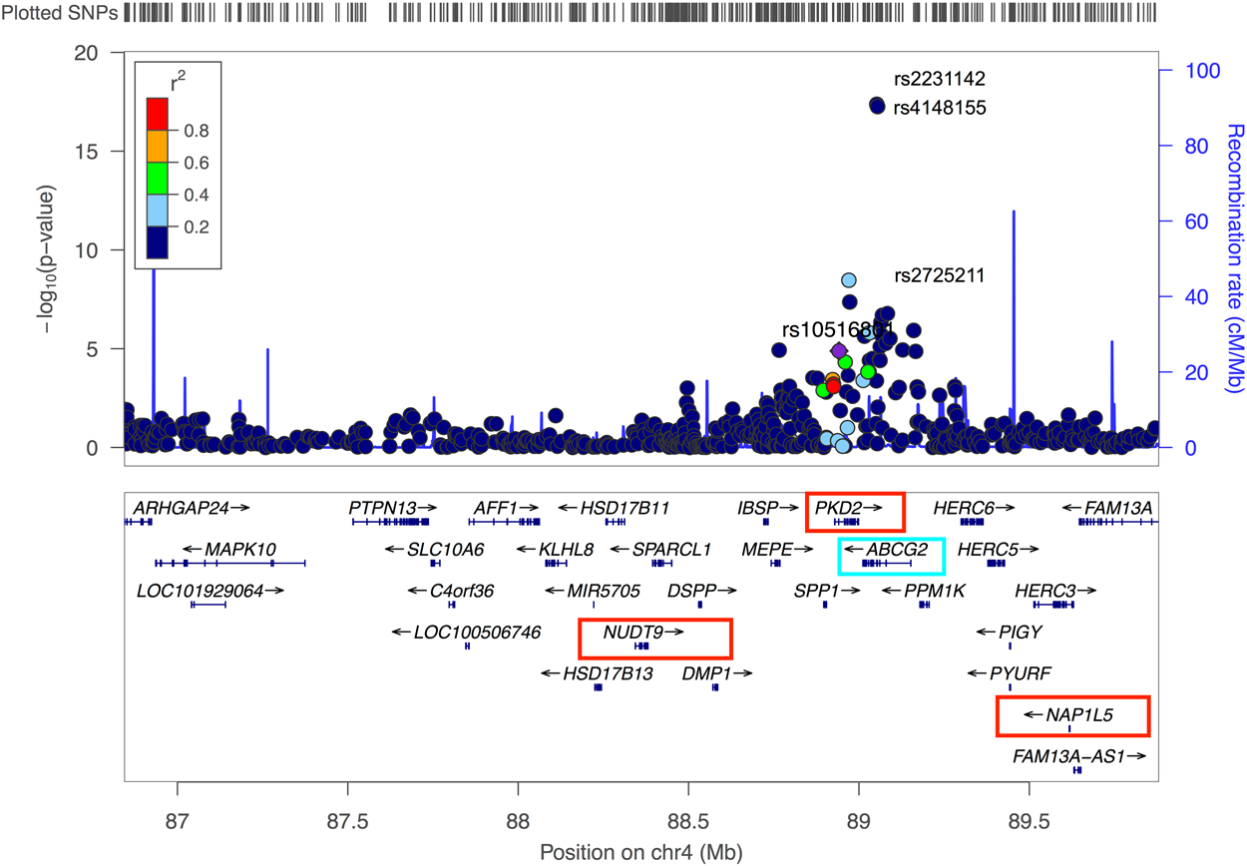
Regional association plot and linkage disequilibrium (LD) on chromosome 4.

We performed a genome-wide association meta-analysis on two Taiwanese cohorts consisting of 758 gout cases and 14166 controls of Han Chinese ancestry. Characteristics of the study subjects are shown in Supplementary Table 4. After performing a standard quality control procedure, we analyzed 373 individuals with gout (cases) and 6721 controls without gout from Taiwan Biobank in the discovery stage. In the discovery stage, we identified 4 SNPs that showed significant association with gout at the genome-wide level (*P* = 5.0 × 10^−8^). (Fig. 1 and Table 1) All of these SNPs are located in previously identified regions on chromosome 4. The only exception that we found was rs2905274 (*P* = *3*.*91* × *10*^*−8*^; OR, 1.87), which was located on chromosome 7. The top-associated SNP in chromosome 4 were rs2231142 (*P* = *4*.*25* × *10*^*−18*^; OR, 2.00) and rs4148155 (*P* = *5*.*49* × *10*^*−18*^; OR, 2.00), which have been mapped to the *ABCG2* gene. We also found that rs2725211 on chromosome 4 was also associated with increased risk of gout (*P* = *3*.*42* × *10*^*−9*^; OR, 1.64), and was located within a genomic region that encodes both the *ABCG2* and PKD2 gene. The regional association plot showed that all the strongly associated SNPs were confined to regions around *ABCG2* and *PKD2* gene (Fig. 2).

**Table 1:** Results of association analyses of gout.

SNP	Chr	Allele 1/2^a^	Stage	Cases				Controls				Additive^b^		Dominant^b^		Recessive^b^		P_het_
				11	12	22	RAF	11	12	22	RAF	P-value	Risk allele OR (95% CI)	P-value	Risk allele OR (95% CI)	P-value	Risk allele OR (95% CI)	
rs2231142	4	T/G	Discovery	82	181	110	0.46	630	2885	3200	0.31	4.25e-18	2.00	5.27e-11	2.18	2.66e-15	3.00	0.9644
			Follow-up	78	197	109	0.46	696	3202	3538	0.31	1.498e-18	1.99	2.86e-13	2.366	2.96e-12	2.65	
			Combined^c^	160	378	219	0.46	1326	6087	6738	0.31	5.06e-35	2.00	1.10e-22	2.27	6.50e-26	2.82	
rs4148155	4	G/A	Discovery	82	181	110	0.46	633	2889	3195	0.31	5.49e-18	2.00	6.27e-11	2.17	3.16e-15	2.99	0.9893
			Follow-up	79	197	109	0.46	702	3204	3536	0.31	8.593e-19	2.00	2.65e-13	2.37	1.29e-12	2.68	
			Combined^c^	161	378	219	0.46	1335	6093	6731	0.31	3.74e-35	2.00	1.21e-22	2.27	3.29e-26	2.83	
rs2725211	4	T/C	Discovery	48	153	172	0.33	386	2418	3900	0.24	3.42e-09	1.64	8.52e-06	1.63	9.38e-09	2.69	0.9417
			Follow-up	45	168	172	0.34	426	2764	4244	0.24	3.78e-09	1.62	1.22e-06	1.69	9.96e-07	2.35	
			Combined^c^	93	321	344	0.33	812	5182	8144	0.24	6.88e-17	1.63	4.75e-11	1.66	5.43e-14	2.52	
rs2905274	7	A/G	Discovery	12	80	279	0.14	44	1027	5640	0.083	3.91e-08	1.87	2.46e-06	1.84	4.19e-06	5.21	0.0041
			Follow-up	5	71	306	0.11	58	1231	6138	0.091	0.26	1.15	0.34	1.14	0.2829	1.69	
			Combined^c^	17	151	585	0.12	102	2258	11778	0.087	1.46e-06	1.50	4.81e-05	1.46	1.42e-05	3.50	

We analyzed 758 gout cases (in the GWAS and in replication) and 14,166 controls (6,721 in the GWAS and 7,445 in replication). Chr., chromosome; RAF, risk allele frequency. ^a^Allele 1, risk allele; allele 2, non-risk allele. ^b^P values and ORs were calculated by logistic regression analysis, with age, gender, and 10 principal components as covariates. Non-risk alleles were considered as references in the three genetic models: additive, 1 versus 2; recessive, 11 versus 12 + 22; dominant, 11 + 12 versus 22. Heterogeneity across the two stages was examined by Cochran Q test under a genetic model which provided the minimum P value in the screening stage. ^c^ORs and P values were calculated using the Mantel-Haenszel fixed-effects model.

In the follow-up GWAS study using 385 independent gout cases and 7,445 controls, we still observed significant associations for the three SNPs on chromosome 4. However, rs2905274 on chromosome 7 failed to replicate. In the combined analysis of the discovery and follow-up cohorts, we identified significant associations for rs2231142 (*P* = *5*.*06* × *10*^*−35*^; OR, 2.00), rs4148155 (*P* = *3*.*74* × *10*^*−35*^; OR, 2.00), and rs2725211 (*P* = *6*.*88* × *10*^*−17*^; OR, 1.63) in the additive model, without any heterogeneity between the two stages.

### Candidate causal SNPs and pathways from the meta-analysis data of GWASs

Utilizing the SNPs *p*-values from the genome-wide association meta-analysis analysis as input, ICSNPathway analysis identified one candidate causal SNP (rs2231142), one gene (ABCG2), and three candidate causal pathways. rs2231142 is not in LD with any SNP, and the candidate causal pathways provide three related hypothetical biological mechanisms of gout: ABC transporters; ATPASE activity coupled; and ATPASE activity coupled to movement of substances (Supplementary Table 5).

### Integrative analysis of eQTL and GWAS results using Sherlok

Through systematic integration of lymphoblastoid eQTL and SNP associations from our discovery GWAS analysis, *PKD2* expression showed the most significant association with gout (LBF = 6.89, *P*_sher_ = 1.08 × 10^−5^) followed by *NUTD9*, *NAP1L5*, *and BRE* (Table 2). In the follow-up analysis, we still observed significant associations for all the genes identified by Sherlock, with the only exception for *BRE*. Interestingly, *PKD2*, *NUTD9 and NAP1L5* are all in the 4q22.1 locus.

**Table 2:** Predicted regulatory genes and SNPs for the risk of gout in lymphocytes.

Gene	SNP	Proximity	Location	Result in Discovery		Result in Follow-up	
				LBF	P*	LBF	*P*
PKD2			4q22.1	6.89	1.08e-05	6.93	8.98e-06
	rs4148155	cis	chr4:89273691	7.02	2.00e-05	7.02	2.00e-05
NUDT9			4q22.1	6.41	2.87e-05	5.79	6.29e-05
	rs10516801	cis		5.62	8.00e-05	6.02	8.00e-05
NAP1L5			4q22.1	5.54	9.52e-05	6.10	4.67e-05
	rs4148155	cis	chr4:89273691	5.62	2.00e-04	5.62	2.00e-04
BRE			2p23.2	5.87	6.11e-05	2.57	5.76e-03
	rs4148155	trans	chr4:89273691	2.66	9.90e-06	2.66	9.90e-06
	rs4129943	trans	chr2:241402488	2.25	2.00e-06	−0.102	2.00e-06

*The gene p-vaule refers to the Sherlock p value, but the SNP p-value refers to the eQTL P value.

One eSNP (rs4148155) showed significant association with both *PKD2* (LBFG = 7.02, *P*_eQTL_ = 2.00 × 10^−5^) and *NAP1L5* expression (LBF = 5.62, *P*_eQTL_ = 2.00 × 10^−4^) and strong evidence for association with gout (*P*_GWAS_ = 5.49 × 10^−18^) (Table 2). We also identified a novel cis eSNP (rs4148155), which showed significant association with of *NUTDT9* (LBF = 5.62, *P*_eQTL_ = 8.00 × 10^−5^) but moderate evidence for association with gout (*P*_GWAS_ = 1.31 × 10^−5^; *P*_*Replication*_ = 2.33 × 10^−6^*; P*_combined_ = 1.34 × 10^−10^).

## Discussion

In this study, we sought to identify novel genetic variations that predisposed individuals to gout among 15,300 Han Chinese residing in Taiwan. From 2 independent cohorts, we found 3 SNPs (rs2231142, rs4148155, and rs2725211) that reached genome-wide statistical significance, and these SNPs are in cis of the *ABCG2* and *PKD2* gene. ICSNPathway analysis identified rs2231142 as the candidate causal SNP, and *ABCG2* located in 4q22.1 as the candidate gene. In order to identify other susceptibility genes exhibiting regulatory function underlying gout, we correlated the signatures of expression data of lymphoblastoid B cell with that of GWASs in gout. We identified three genes, which were significantly associated with the risk of gout (*PKD2*, *NUTD9*, *and NAP1L5*), with *NUTD9*, and *NAP1L5* reported at the first time.

Previous studies have reported polymorphism in *ABCG2* to be associated with gout in several populations, such as, European Americans, African Americans, Mexican Americans, Americans Indians, German, Japanese and Han Chinese^9,10,23–25^. The rs2231142 (Arg141Lys) genetic variant at *ABCG2* is a common missense genetic variants, and meta-analysis of existing study found the rs2231142 Arg141Lys carriers was associated with 1.73 fold increased susceptibility of gout. It has been reported that the Arg141Lys variant of ABCG2 causes instability in the nucleotide-binding domain of ABCG2, and lead to decreased surface expression and function of ABCG2^26^. As a result, rs2231142 Arg141Lys carriers have decreased uric acid excretion through both the kidney and the gut with the potential for hyperuricemia. Besides leading to hyperuricemia, ABCG2 dysfunction was also found to be involved in subsequent steps in gout formation. Knock down of ABCG2 by siRNA led to gouty inflammation involving the release of IL-8 upon MSU crystals-stimulation^23^. In addition, in a Taiwanese study of Han Chinese with gout, rs2231142 Arg141Lys carriers were associated with 1.51 fold increased risk of tophi^27^.

Besides the rs2231142 variant, this study also found that the rs4148155 variant of ABCG2 was associated with gout. This is likely due to the fact that rs2231142 and rs4148155 are completely in LD in the Han Chinese. The rs4148155 genetic variant was reported to be an intron variant of ABCG2, and was also found to be associated with uric acid formation in both Han Chinese and Japanese population^28,29^. Interestingly, in our Sherlock analysis using lymphocytes, the rs4148155 variant was associated with the eQTL of *PKD2*, and *NAP1L5*. *ABCG2*, *PKD2*, *NAP1L5* are all located in the 4q22.1 region, and we hypothesize a cis acting epistatic interactions between these genes. In fact, a previous study in Han-Chinese found also found a positive correlation between *ABCG2* mRNA expression and *PKD2* mRNA expression^30^. Currently, the biological mechanism on how ABCG2 interacts with PKD2/NAP1L5 in the pathogen of gout is unclear. But there are strong clinical and genetic reports linking gout and PKD2. PKD2 encodes Polycystin-2, which is the protein mutated in autosomal dominant polycystic kidney disease (ADPKD)^31^. It is well recognized that patients with ADPKD develop renal failure and progress to hyperuricemia and increased risk of gout^32,33^. In addition, our Sherlock analysis validated Genecards’ report that PKD2 is expressed in lymphocytes. The role of lymphocytes related to gout development has been well recognized, but it unclear how ABCG2 interact with PKD2 in the inflammation stage of gout^34,35^. Future research into the functional role of PKD2 in lymphocytes may also help explain why not all ADPKD patients develop gout. As for NAP1L5, it has been implicated in IL-8 release, and IL-8 release has been found to be an important activator for monosodium urate crystal‐induced arthritis^36–38^. Interestingly, siRNA knock down of ABCG2 also increases IL-8 release^23^.

Results of this study have to be interpreted in light of several limitations. First, this was a case-control study conducted in a Han Chinese population residing in Taiwan. Future investigations using other populations will be critical to clarify whether these newly identified susceptible genes are shared in other populations. Second, this study focused on only common SNPs and did not consider the contributions of rare variants. Future studies on rare variants should also be conducted to fully understand the role of rare variants in the pathogenesis of gout. Third, this study did not conduct a functional study to identify the causal variant for gout disease, and functional study should be conducted by follow-up studies.

In summary, we have identified several genetic loci related to gout using GWAS in the Han Chinese population in Taiwan. We performed single-marker as well as pathway analyses to identify genetic associations with gout. The rs2231142 (Arg141Lys) genetic variant at ABCG2 was identified to be the causal SNP, but this SNP was also found to be in complete LD with rs4148155. In addition, we conducted Sherlock analysis to identify susceptibility genes in with regulatory function in gout, and identify three genes, which were significantly associated with the risk of gout (*PKD2*, *NUTD9*, and *NAP1L5*). To conclude, the results of our study may contribute to the understanding of the genetic causes of gout, and future studies are needed to confirm and explore the role of NUTD9, and NAP1L5 in the pathogenesis of gout.

## Supplementary information

Supplementary info

## Data Availability

The data used in this study are available for purchase from Taiwan Biobank. To gain access, interested individuals should contact “biobank@gate.sinica.edu.tw”. The GWAS data will be deposited in the GWAS catalog upon manuscript acceptance by a peer-reviewed journal.
