# Supplementary material for: Integrative Genome-Wide Association Studies of eQTL and GWAS Data for Gout Disease Susceptibility
Source: Sci Rep. 2019 Mar 21;9:4981. doi: 10.1038/s41598-019-41434-4 (PMC6428872; doi:10.1038/s41598-019-41434-4)
Supplement: Supplementary file 1 — Supplementary info [file 41598_2019_41434_MOESM1_ESM.docx]

**Integrative Genome-Wide Association Studies of eQTL and GWAS Data for Gout Disease Susceptibility- Supplementary Data**

Meng-tse Gabriel Lee PhD, Tzu-Chun Hsu BSc, Shyr-Chyr Chen MD EMBA, Ya-Chin Lee BS, Po-Hsiu Kuo PhD, Jenn-Hwai Yang PhD, Hsiu-Hao Chang MD, PhD, Chien-Chang Lee MD, ScD.

Supplementary Table 1. Quality control details for the GWAS and replication participants.

|  | Original samples | Sex mis-match | Call rate <97% | Kinship > 0.8 | Population stratification | Number at end of QC |
| --- | --- | --- | --- | --- | --- | --- |
| **Discovery sample** | | | | | | |
| Total | 7300 | 0 | 0 | 206 | 0 | 7094 |
| **Follow-up sample** | | | | | | |
| Total | 8000 | 18 | 62 | 90 | 0 | 7830 |

Supplementary Table 2. Quality control details for the SNPs

|  | Original samples | HWE *P*< .000001 | Total sample call rate >0.9 | MAF<0.01 | Number at end of QC |
| --- | --- | --- | --- | --- | --- |
| **Discovery sample** | 631941 | 4456 | 17302 | 2508 | 607675 |
| **Follow-up sample** | 634232 | 6316 | 13973 | 4572 | 621874 |

Supplementary figure 1. Population stratification analysis for discovery sample. (A) Scree plot of eigenvalues for 20 principal components. (B) Key principal components.

A


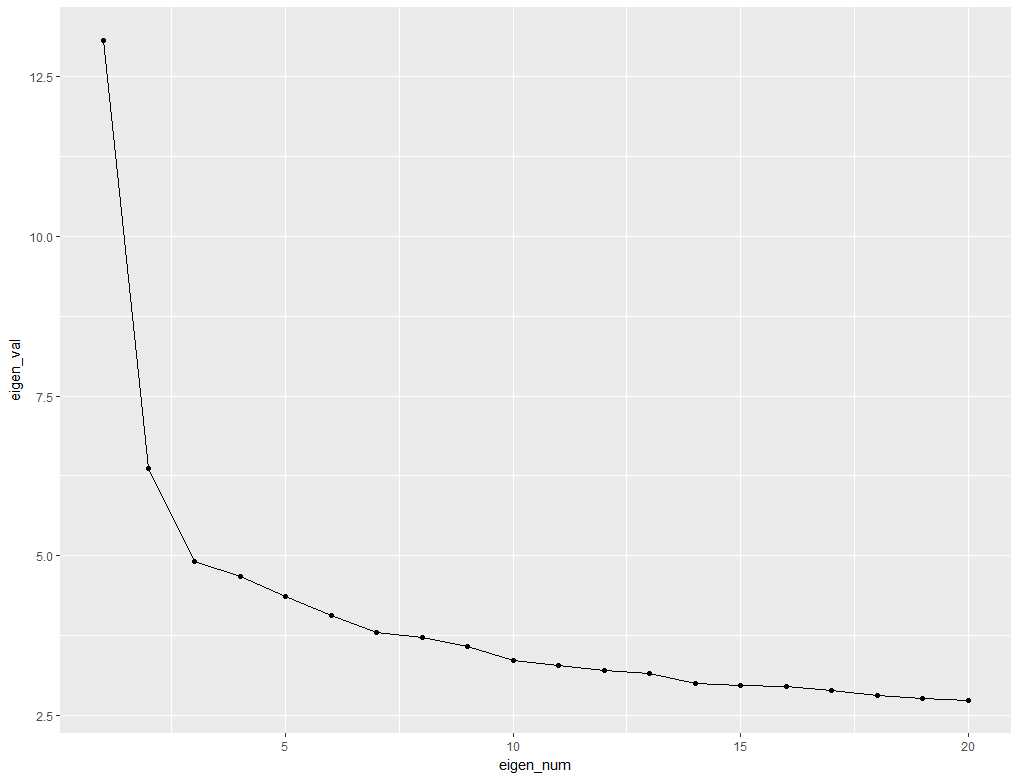


B


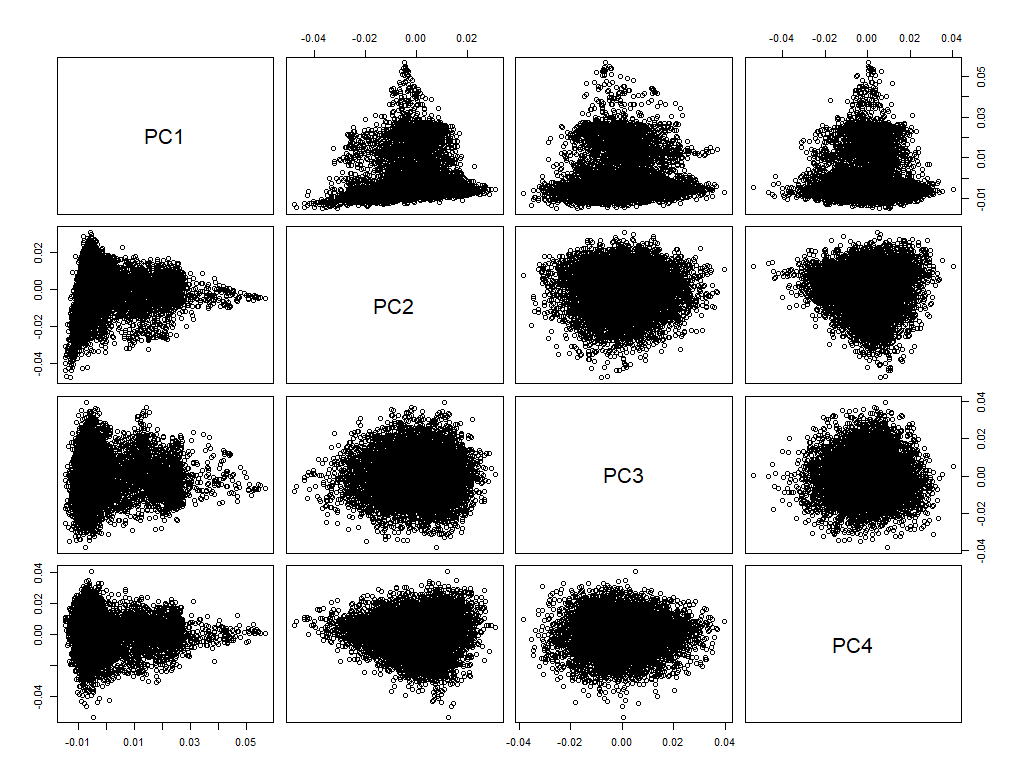


Supplementary Table 3. Power analysis using 758 gout cases and 14166 controls.

| Allele frequencies | 0.05 | 0.10 | 0.20 | 0.30 | 0.40 | 0.55 | 0.75 | 0.85 |
| --- | --- | --- | --- | --- | --- | --- | --- | --- |
| OR |  |  |  |  |  |  |  |  |
| 1.10 | 0% | 0% | 0% | 0% | 0% | 0% | 0% | 0% |
| 1.20 | 0% | 0% | 0.4% | 1.2% | 1.7% | 1.6% | 0% | 0% |
| 1.30 | 0.1% | 0.9% | 7.5% | 16.6% | 21.7% | 18.8% | 4.8% | 0.8% |
| 1.40 | 0.6% | 6.6% | 35.8% | 57.4% | 65% | 58.2% | 20.9% | 4.2% |
| 1.50 | 2.6% | 23.8% | 73% | 89% | 92.2% | 87.6% | 46.7% | 12.2% |
| 1.60 | 8.4% | 50.9% | 93.4% | 98.5% | 99% | 97.7% | 70.7% | 24.7% |
| 1.70 | 19.8% | 76% | 99% | 99.9% | 99.9% | 99.7% | 86.3% | 39.5% |
| 1.80 | 36.2% | 91.2% | 99.9% | 100% | 100% | 100% | 94.2% | 54% |
| 1.90 | 54.7% | 97.5% | 100% | 100% | 100% | 100% | 97.7% | 66.4% |
| 2.00 | 71.4% | 99.4% | 100% | 100% | 100% | 100% | 99.1% | 76.1% |

Power analysis is conducted using genetic association study (GAS) power calculator at the significance threshold of P =5.0 X 10^-8^. Power figures at representative and relevant ORs ( 1.1 to 2.0) and allele frequencies (0.05 to 0.85) are displayed.

Supplementary Table 4. Basic characteristics of study subjects.

|  | **Case** | **Control** |
| --- | --- | --- |
| **Discovery sample** |  |  |
| Number of samples | 373 | 6721 |
| Male (%) | 362(97.05%) | 3163(47.06%) |
| Age at sampling, y*ear, SD* | 52.52±10.66 | 49.64±11.36 |
| **Follow-up sample** |  |  |
| Number of samples | 385 | 7445 |
| Male (%) | 362(94.02%) | 3604(48.41%) |
| Age at sampling, y*ear, SD* | 49.82±10.27 | 47.63±10.83 |

Supplementary figure 2. Quantile-Quantile plot of genome-wide associations for discovery sample. (A) armitage trend test is conducted; (B) additive logistic regression with PCA adjustment is conducted.

(A)


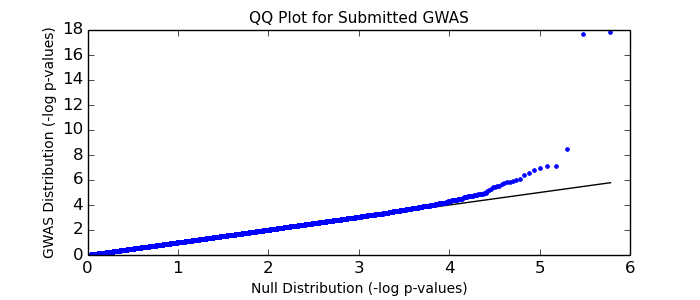


(B)


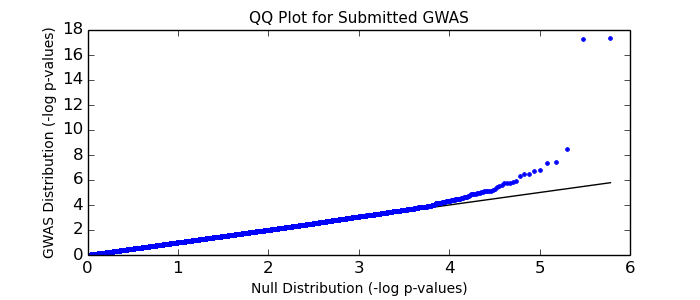


Supplementary Table 5. Candidate causal pathways of gout from the meta-analysis data of GWAS.

| **Index** | **Candidate causal pathway** | **Nominal *p*** | **FDR** |
| --- | --- | --- | --- |
| 1 | ABC transporters | 0.005 | 0.021 |
| 2 | ATPASE activity coupled | 0.014 | 0.023 |
